# Supplementary material for: An Extracellular Redox Signal Triggers Calcium Release and Impacts the Asexual Development of Toxoplasma gondii
Source: Front Cell Infect Microbiol. 2021 Aug 10;11:728425. doi: 10.3389/fcimb.2021.728425 (PMC8382974; doi:10.3389/fcimb.2021.728425)
Supplement: Supplementary Table 1 — List of Toxoplasma gondii genes related to redox sensing systems and their primary location based on hyperLOPIT datasets. PITH, proteasome-interacting thioredoxin; ER, endoplasmic reticulum; PM, plasma membrane; GSH, glutathione; NA, not applicable (location not predicted). [file Table_1.pdf]

Gene ID (ToxoDB)	Gene annotation	Predicted localisation	Publication indicating redox function
TGME49_204480	thioredoxin domain-containing protein	apicoplast	no
TGME49_209950	thioredoxin domain-containing protein	apicoplast	no
TGME49_216510	thioredoxin, putative	mitochondria-membrane	no
TGME49_218530	PITH domain-containing protein	cytosol	no
TGME49_240500	PITH domain-containing protein	19S proteasome	no
TGME49_224060	thioredoxin, putative	golgi	no
TGME49_247350	thioredoxin domain-containing protein	ER	no
TGME49_269950	thioredoxin domain-containing protein	dense granules	no
TGME49_201800	thioredoxin domain-containing protein	mitochondria-membrane	no
TGME49_247660	thioredoxin domain-containing protein	NA	no
TGME49_255480	thioredoxin domain-containing protein	NA	no
TGME49_266620	thioredoxin domain-containing protein	ER	yes(1)
TGME49_310770	hypothetical protein / ATrx2	apicoplast	yes(2)
TGME49_265510	hypothetical protein	19S proteasome	no
TGME49_270120	thioredoxin-like protein, TLP1	mitochondria-soluble	no
TGME49_289180	thioredoxin domain-containing protein	mitochondria-soluble	no
TGME49_290260	PITH domain-containing protein	cytosol	yes(1)
TGME49_291810	thioredoxin domain-containing protein	NA	no
TGME49_293870	thioredoxin	cytosol	no
TGME49_271760	seryl-tRNA synthetase (SeRS2)	ER	no
TGME49_270140	splicing factor DIM1, putative	cytosol	no
TGME49_308050	phosducin domain-containing protein	NA	no
TGME49_309730	thioredoxin reductase	cytosol	yes (3)
TGME49_247025	PITH domain-containing protein	NA	no
TGME49_258826	thioredoxin domain-containing protein	ER	no
TGME49_312110	thioredoxin domain-containing protein	apicoplast	yes(4)
TGME49_225060	thioredoxin domain-containing protein	nucleus-chromatin	no
TGME49_211680	protein disulfide-isomerase	ER	yes(5)
TGME49_218470	protein disulfide-isomerase, putative	NA	no
TGME49_225790	thioredoxin-like-fold domain-containing protein	cytoskeleton	no
TGME49_232410	thioredoxin-like-fold domain-containing protein	cytoskeleton	no
TGME49_238040	protein disulfide-isomerase domain-containing protein	ER	no
TGME49_249270	protein disulfide isomerase-related protein (provisional), putative	apicoplast	no
TGME49_226800	GSH-synthase domain-containing protein	nucleolus	
TGME49_238070	glutaredoxin domain-containing protein	cytosol	no
TGME49_277790	glutaredoxin domain-containing protein	PM-peripheral	no
TGME49_304660	glutaredoxin domain-containing protein	mitochondria-soluble	no
TGME49_247580	glutaredoxin domain-containing protein	rhoptries	no
TGME49_227150	glutaredoxin domain-containing protein	apicoplast	no
TGME49_279400	glutaredoxin domain-containing protein	cytosol	no
TGME49_268730	glutaredoxin domain-containing protein	mitochondria-soluble	no
TGME49_246920	glutathione reductase	19S proteosome	no
TGME49_306030	glutathione s-transferase, n-terminal domain containing	ER	no
TGME49_249630	glutathione s-transferase, N-terminal domain-containing protein	mitochondria-soluble	no
TGME49_281630	lactamase-b domain-containing protein	nucleolus	no
TGME49_219130	NADPH-glutathione reductase	apicoplast	no
TGME49_227100	glutaredoxin domain-containing protein	NA	no
TGME49_285920	hypothetical protein	NA	no
TGME49_309210	thioredoxin domain-containing protein / peroxiredoxin 6, putative	cytosol	no
TGME49_230410	thioredoxin domain-containing protein / peroxiredoxin PRX3	mitochondria-soluble	yes(6, 7)
TGME49_266130	glutathione peroxidase / peroxiredoxin PRX2	NA	yes(7)
TGME49_217890	peroxiredoxin / alkyl hydroperoxide reductase	nucleus	yes(8)
TGME49_286630	peroxiredoxin / redoxin domain-containing protein	apicoplast	yes(8)
TGME49_266120	glutathione peroxidase / thioredoxin-dependent peroxidase TPX1	PM-peripheral	yes(9)
TGME49_316330	superoxide dismutase SOD2	mitochondria-soluble	yes(7)
TGME49_232250	catalase	cytosol	yes(7, 10, 11)
TGME49_316190	superoxide dismutase SOD3	NA	yes(7)
TGME49_316310	superoxide dismutase	cytosol	yes(12)
