# Supplementary material for: An Extracellular Redox Signal Triggers Calcium Release and Impacts the Asexual Development of Toxoplasma gondii
Source: Front Cell Infect Microbiol. 2021 Aug 10;11:728425. doi: 10.3389/fcimb.2021.728425 (PMC8382974; doi:10.3389/fcimb.2021.728425)
Supplement: Supplementary Table 2 — List of primers used to generate plasmid and check sequence/integration in this study. [file Table_2.pdf]

Primer name	Primer number	Sequence (5'-3')
Fw-GRX1-roGFP2	1	ctcgttggcattttttcttgatggctcaagagtttgtgaac
Rv-GRX1-roGFP2	2	agtgagcacaacggtgattattacttgtacagctcgtccatg
Fw-GRA8-GRX1-roGFP2	3	ctcgttggcattttttcttgatggctttaccattgcgtgttt cggccacggtgttcgtggtcttcgctgtctttggtgtagct cgcgccatggggaaggtggttgtgttc
Fw-GRX1ser23-26	4	ccagcccaccagcccgtacagcag
Rv-GRX1ser23-26	5	atgaacacaaccaccttcccagg
Rv-GRA8-GRX1ser23-26	6	atgaacacaaccaccttccccat
Fw-pTUB8 sequence	7	cagaagacatccaccaaacg
Rv-pTUB8 sequence	8	gctgcgaaacactcctatttag
Fw-jRCaMP1b	9	actttcgtcgtagtcttaatctacttcgctgtcatcatttgtac
Rv-jRCaMP1b	10	tccaggaccaatgctgcagaacgagcttg
Fw-5'UTR of GRA1	11	tgctcaccatcttgcttgatttcttcaaag
Rv-5'UTR of GRA1	12	ccggactacgcgtagttaattcgaaggctgtagtactg
Fw-GFP-T2A-fusion	13	tctgcagcattggtcctggattttcttctacatc
Rv-GFP-T2A-fusion	14	atcaagcaagatggtgagcaagggcgag
Fw-HXGPRT cassette	15	caagaatccccactccaagcatgcaggcgcagctggagaatggcca gaggcaggcatc
Rv-HXGPRT cassette	16	caaccggctgcatgcacgacgaagacatgtgcacctaaagccgcggaa gatccgatcttg
Rv-pSAg1::CAS9-U6::sg1/2 CDPK3	17	aacttgacatccccatttac
Fw-pSag1::Cas9-U6::sg1 CDPK3	18	gacgccagcctcgaaaaggggttttagagctagaaatagc
Fw-pSag1::Cas9-U6::sg2 CDPK3	19	tcagtccgtagttgggacaggttttagagctagaaatagc
Fw-sg1CDPK3	20	gtaatacgactcactatagggcgaattgggtacccaagtaagc agaagcacgct
Rv-sg1CDPK3	21	gtaaaagcttatcgataccgtcgacctcgagaattaaccctc actaaagg
Fw-5'HXGPRT cassette at CDPK3 locus	22	ggagtcacgcctgagtttga
Rv-5'HXGPRT cassette at CDPK3 locus	23	ggcctacgtgacttgctgat
Fw-3'HXGPRT cassette at CDPK3 locus	24	cttcaatgggtttggacgcc
Rv-3'HXGPRT cassette at CDPK3 locus	25	gaacaaagggggtcggtcat
Fw-CDPK3 locus absent check	26	gcgcgttctcaggatgttcgt
Rv-CDPK3 locus absent check	27	cagtgtatctgcaacaaccaga
