# Supplementary figures and images for: An Extracellular Redox Signal Triggers Calcium Release and Impacts the Asexual Development of Toxoplasma gondii
Source: Front Cell Infect Microbiol. 2021 Aug 10;11:728425. doi: 10.3389/fcimb.2021.728425 (PMC8382974; doi:10.3389/fcimb.2021.728425)

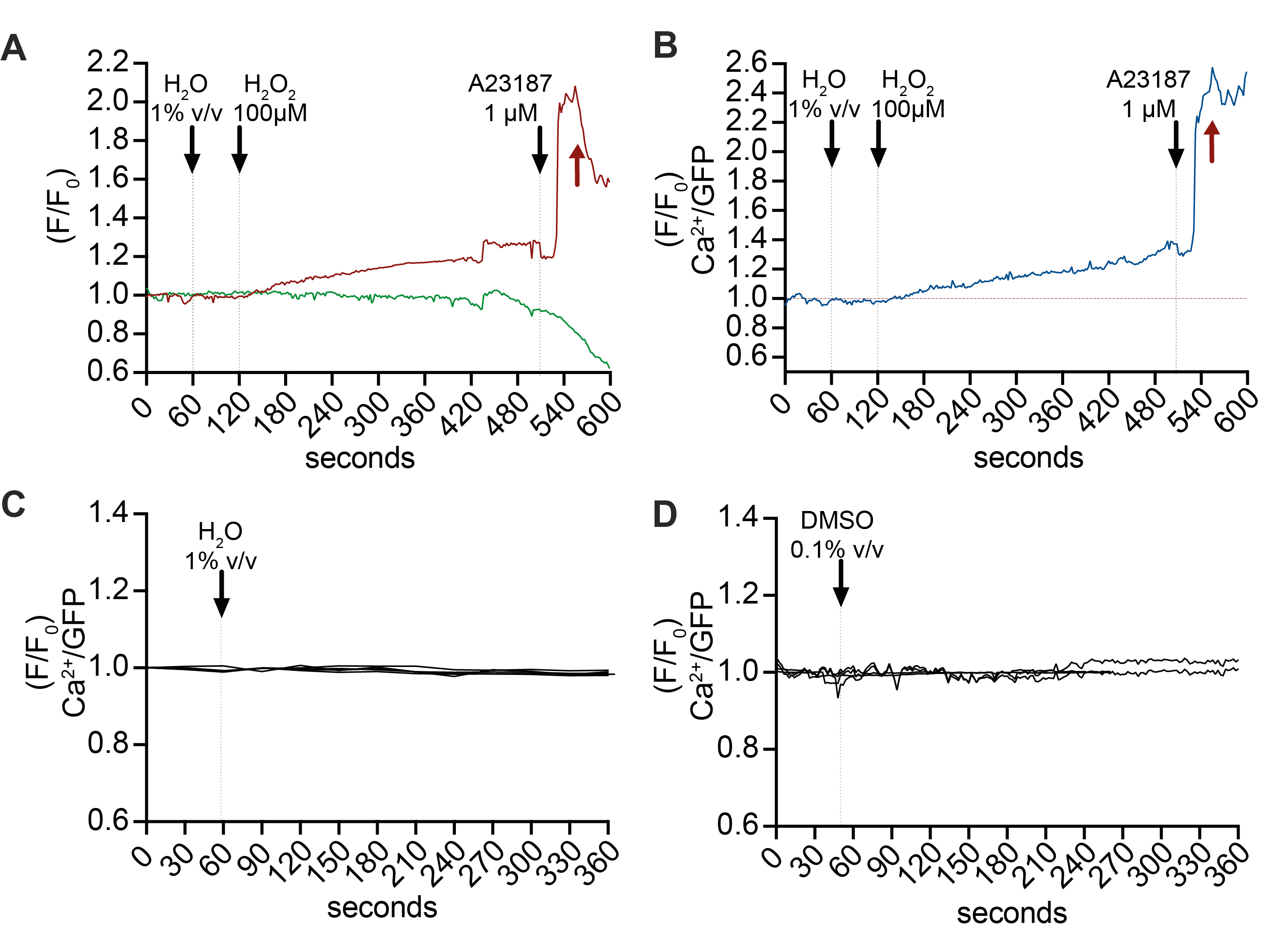

Supplement: Supplementary Figure 1 — Fluorescence tracking of Ca2+ signal and GFP movement on RH-GFP-t2a-jRCaMP1b parasites within host cell. (A, B) Representative trace of parasite vacuoles following treatment with H2O2 and ionophore A23187. (A) The graph presents the independent GFP trace (green) and Ca2+ signal from jRCaMP1b sensor (red). Note the parasite movement at 420s. (B) Graph of the Ca2+ signal is normalized to GFP to minimize artefact on the Ca2+ measurements due to parasite movement. Red arrow indicates the moment of parasite egress. (C) Water, the vehicle solvent for H2O2, does not mobilize Ca2+. The graph displays the trace of three independent vacuoles from the same field of view. Data are representative of 15 infected vacuoles from four independent experiments. (D) DMSO, the vehicle solvent for ionophore A23187, does not mobilize Ca2+. The graph displays the trace of four independent infected vacuoles from the same field of view. Data are representative of 12 rosettes from three independent experiments. (A–D) black arrows indicate the time of drug/solvent addition. [file Image_1.png]

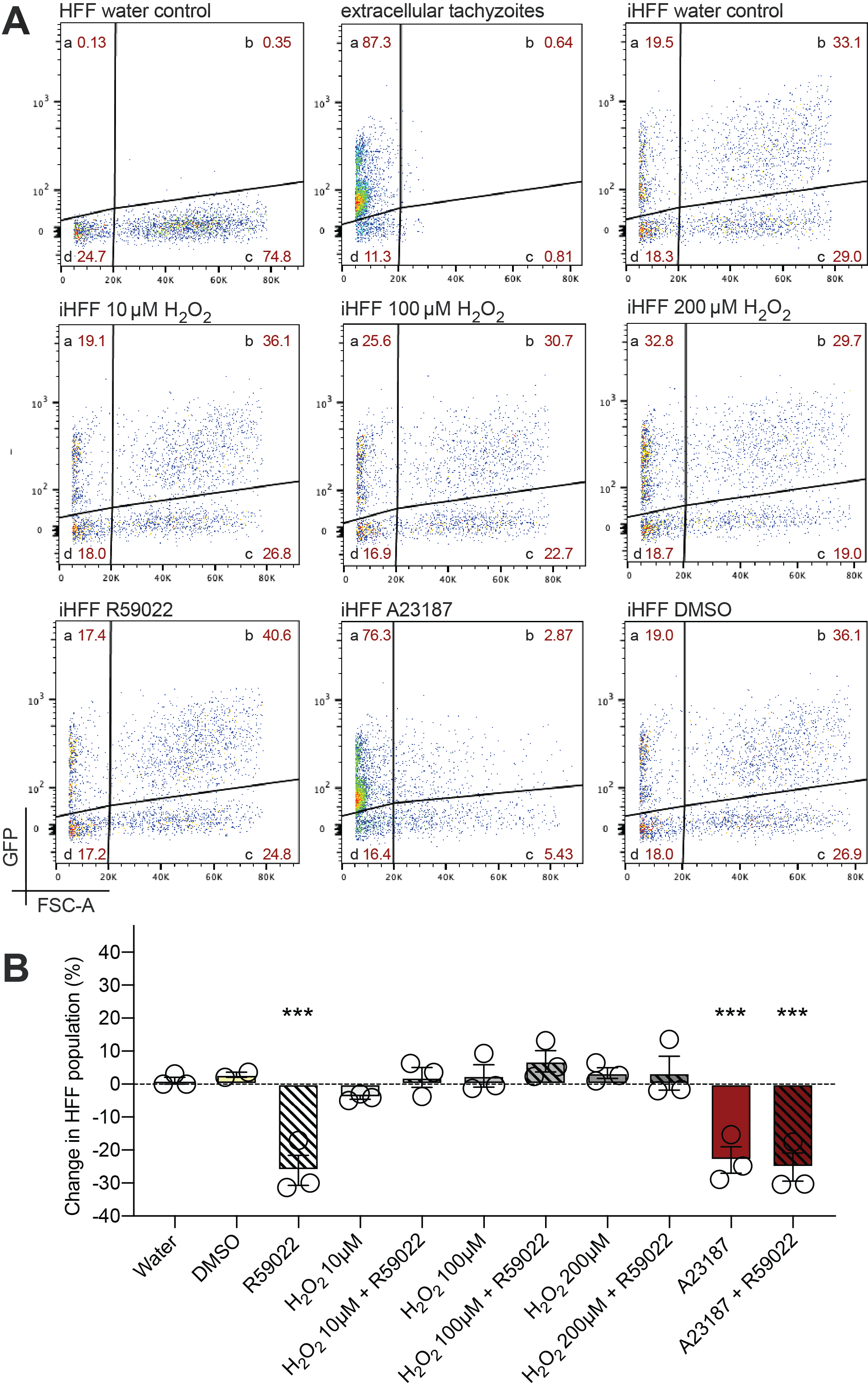

Supplement: Supplementary Figure 2 — Quantification of RH-GFP-T2A-jCamP1b parasite egress by flow cytometry. (A) Representative gating using GFP (Blue laser, 488nm, filter 530/30) against Forward scatter (FSC-A). HFF water control: uninfected human foreskin fibroblast (HFF) are mainly localised in quadrant c. Lysed tachyzoites: free RH-GFP-T2A-jCamP1b parasites are mainly localised in quadrant a. iHFF water control: infected host cells are detected in quadrant b. By using the GFP it is possible to distinguish free fluorescent parasite from iHFF in a population and assess the egress rates throughout different treatments. (B) Effect of drug incubation on non-infected HFF. The graph presents a change on event number within the non-fluorescent HFF gate. Data represent the mean ± SEM of three independent experiments (except for DMSO treatment that has two independent experiment), six technical replicates on each. Significance was calculated using one-way Anova, Bonferroni’s multiple comparisons P value ***< 0.001. [file Image_2.png]

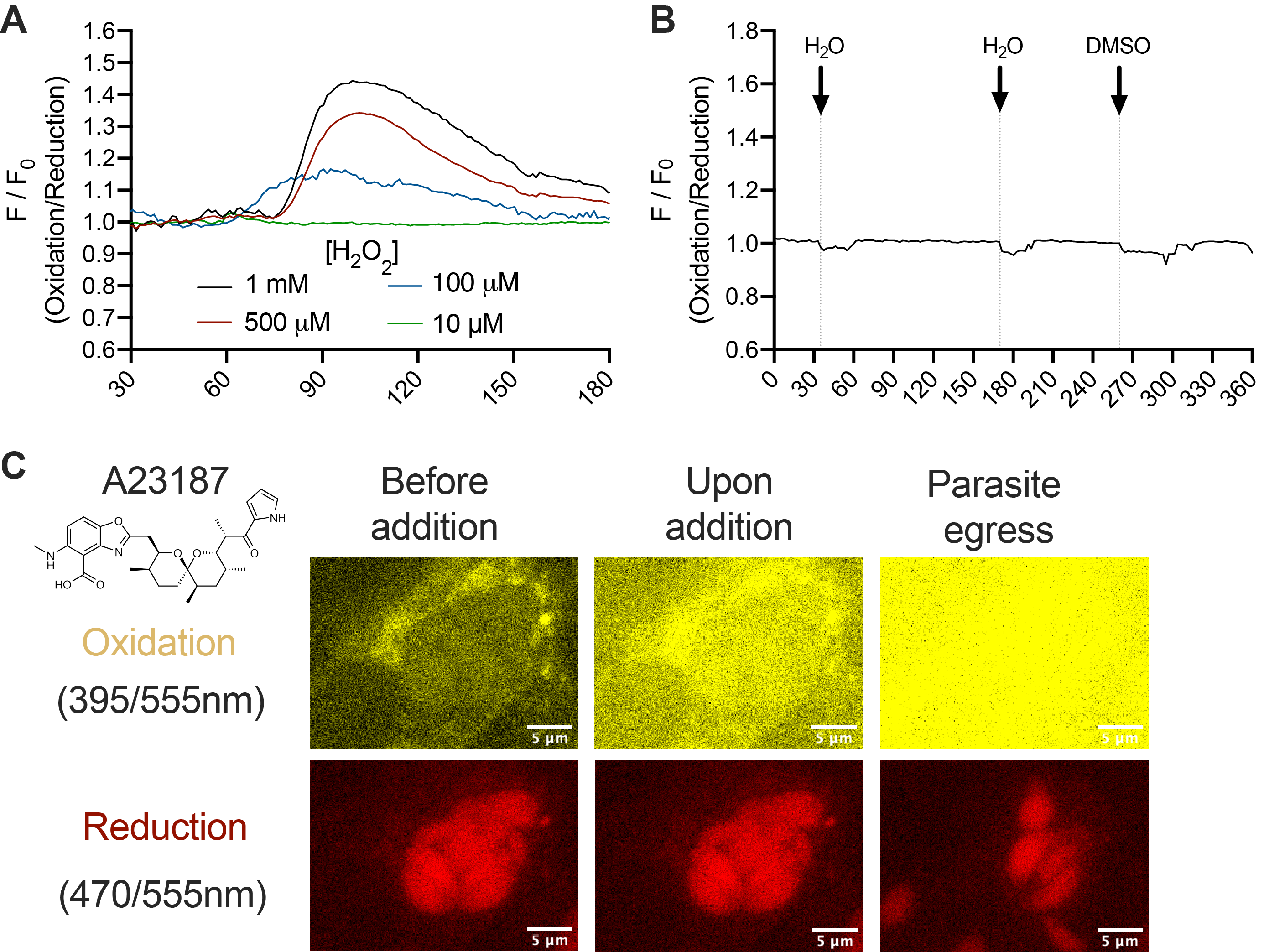

Supplement: Supplementary Figure 3 — Exploring the redox sensitivity of RH-GRX1-roGFP2 parasites to H2O2. (A) Tracking the GSH/GSSG change upon different concentration of H2O2. Among the concentration tested, 10 μM was the only one that did not induce a change in redox within the parasite cytosol. Data are representative of five infected vacuoles from each concentration, one independent experiment. (B) Water (vehicle control for H2O2) and DMSO (vehicle control for A23187) do not trigger change in GSH/GSSG. Data are representative of 12 vacuoles from three independent experiments. (C) Autofluorescence effect of A23187 drug on oxidation channel makes this ionophore unsuitable for ratiometric analyses of GRX1-roGFP2 sensor. Widefield microscope imaging depicting an egress induce by A23187. The structure of A23187 is shown. [file Image_3.png]

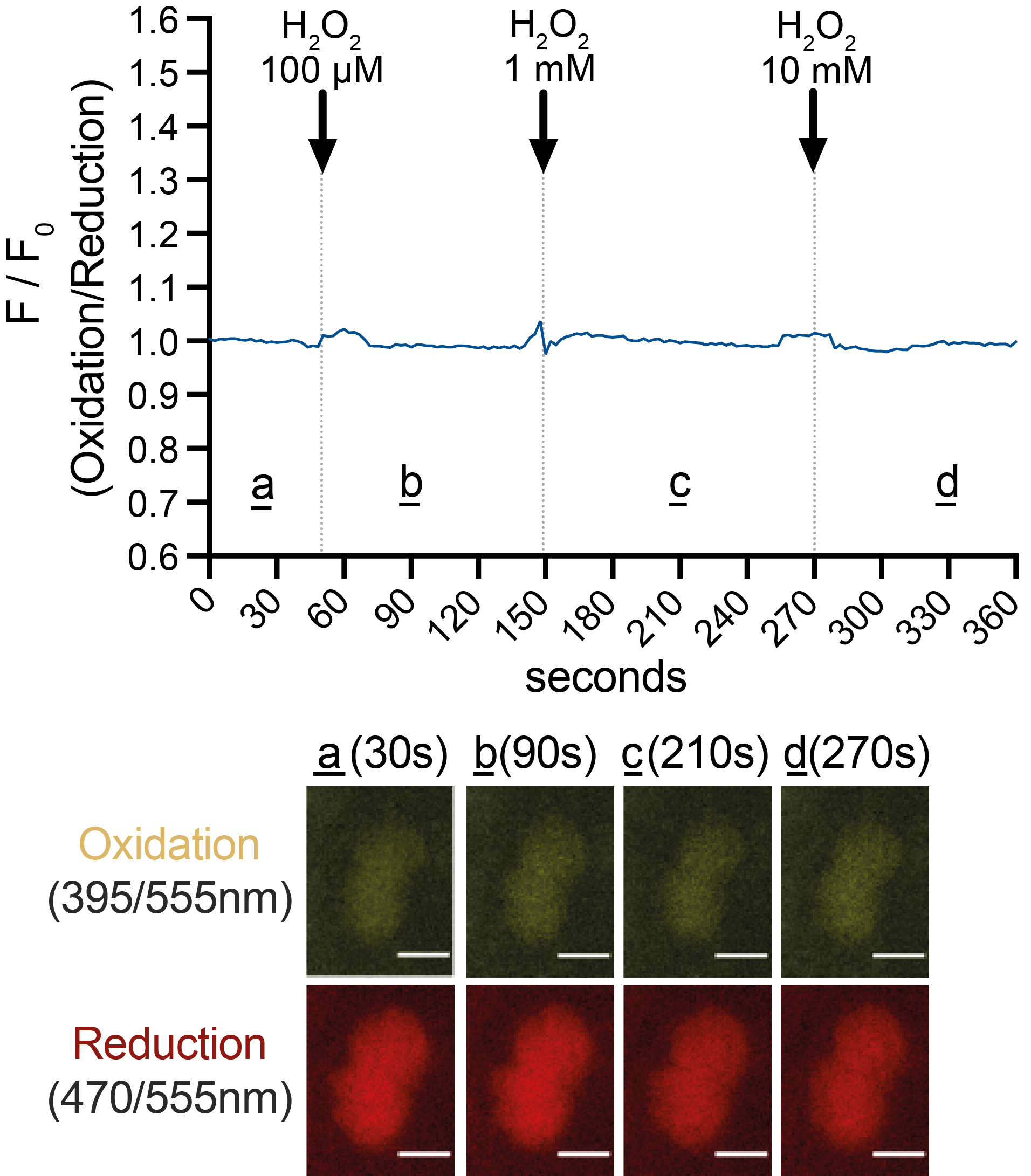

Supplement: Supplementary Figure 4 — Inactivation of the catalytic domain of glutaredoxin 1 makes the GRX1-roGFP2 redox sensor insensitive to changes in GSH/GSSH. Parasite expressing GRX1ser-roGFP2 do not display fluorescent changes in either channel (reduction or oxidation) upon addition of H2O2. Data are representative of 12 infected vacuole, three independent experiments. Black arrows indicate the time of H2O2 addition. [file Image_4.png]

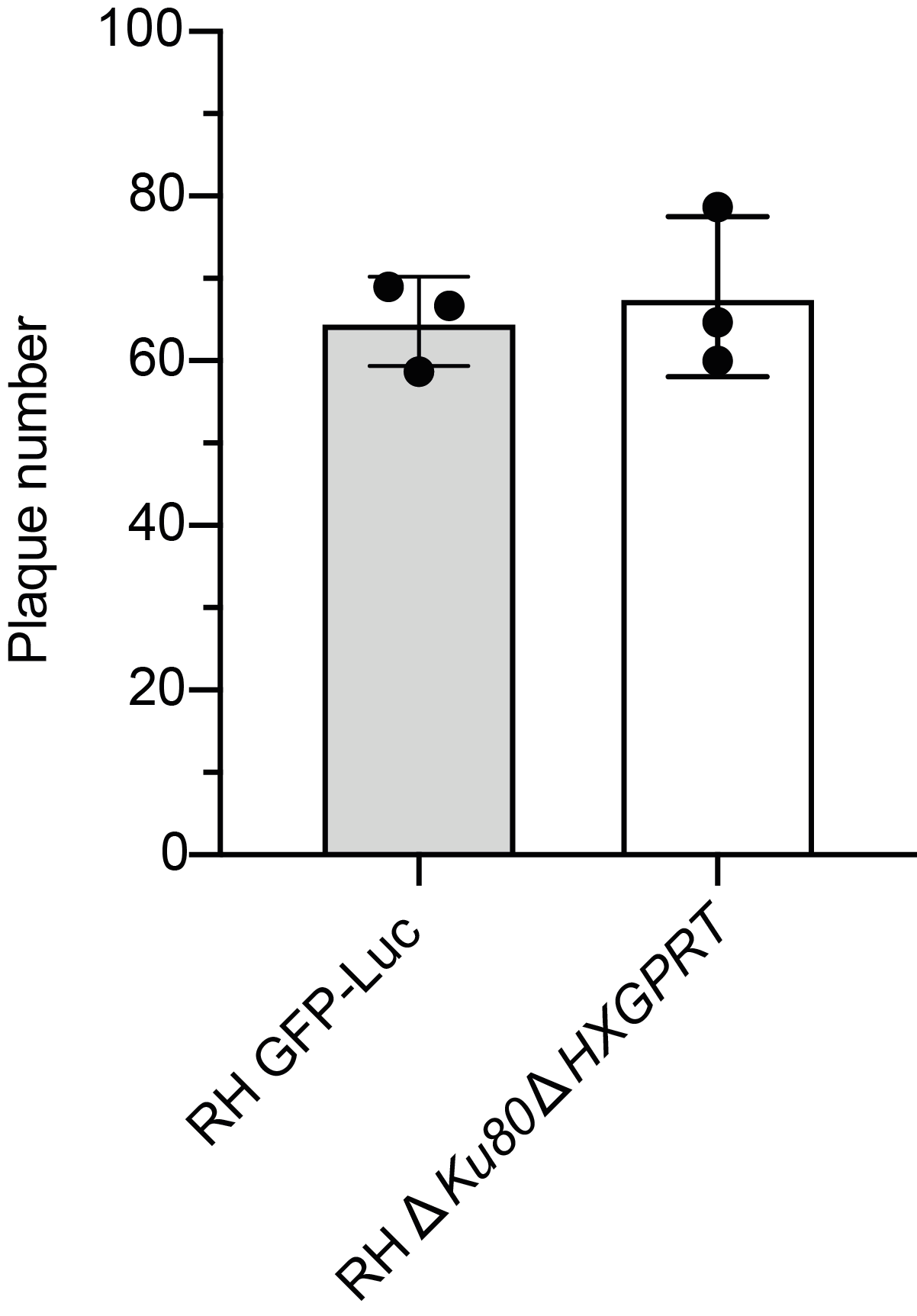

Supplement: Supplementary Figure 5 — Toxoplasma parasites RH-GFP-Luc and RHΔku80ΔHXGPRT plaque with similar efficiency. Bar graphs presenting plaque count data from a six-day plaque assay. The data presented is from three independent biological experiments, each with three technical replicates. No statistically significant difference was found between the two strains using two-tailed paired Student t test. [file Image_5.png]

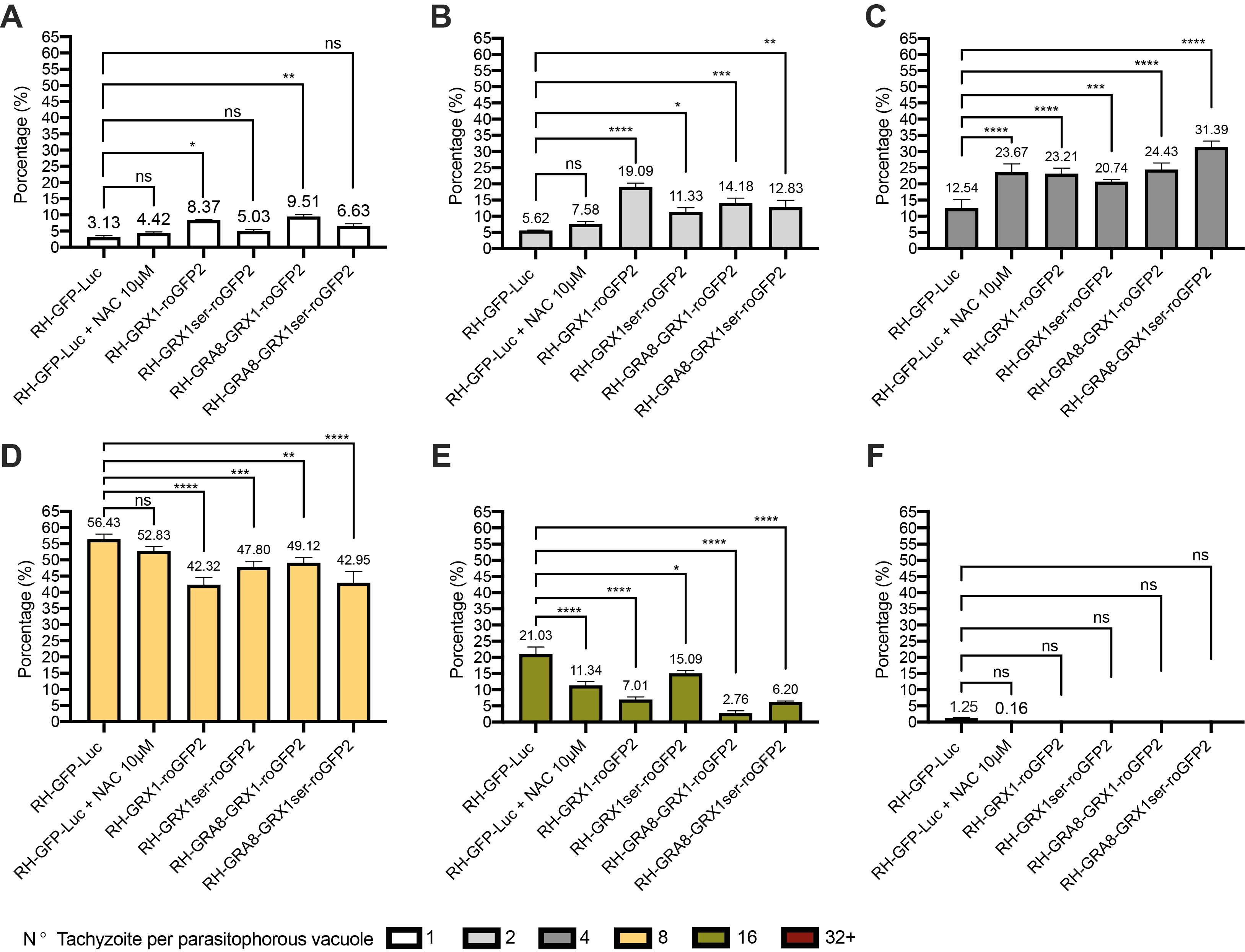

Supplement: Supplementary Figure 6 — Effect of redox sensors on T. gondii parasite growth. Each graph presents parasites/vacuole counts after 20 hours of intracellular growth. The mean values are displayed over each bar for three independent experiments. (A) Percentage (%) of vacuoles contain one parasite. (B) % of vacuoles with two parasites. (C) % of vacuoles with four parasites. (D) % of vacuoles with eight parasites. (E) % of vacuoles with 16 parasites. (F) % of vacuoles with more than 32 parasites. Significance was calculated using two-way Anova, Bonferroni’s multiple comparisons test. P values: *< 0.05; **< 0.01, ***< 0.001 and ****< 0.0001. [file Image_6.png]
